# Estimating the number of probable new SARS-CoV-2 infections among tested subjects from the number of confirmed cases

**DOI:** 10.1186/s12874-023-02077-2

**Published:** 2023-11-17

**Authors:** YM Diarra, PM Wimba, PB Katchunga, J Bengehya, B Miganda, M Oyimangirwe, L Tshilolo, SM Ahuka, J Iwaz, JF Étard, R Écochard, P Vanhems, M Rabilloud

**Affiliations:** 1 Université de Lyon, Lyon, France; 2 Université Claude Bernard Lyon 1, Villeurbanne, France; 3 Service de Biostatistique-Bioinformatique, Pôle Santé Publique, Hospices Civils de Lyon, Lyon, France; 4 Laboratoire de Biométrie et Biologie Évolutive, Équipe Biostatistique-Santé, CNRS UMR 5558, Villeurbanne, France; 5 Université Officielle de Bukavu, Democratic Republic of the Congo, Bukavu, Congo; 6 Cliniques Universitaires de Bukavu, Democratic Republic of the Congo, Bukavu, Congo; 7 Centre International de Recherche en Infectiologie (CIRI), INSERM U1111-CNRS UMR 5308, Lyon, France; 8 Bureau Information Sanitaire, Division provinciale de la Santé Sud-Kivu, Democratic Republic of the Congo, Bukavu, Congo; 9 Université Officielle de Mbujimayi (UOM), Mbuji-Mayi, Democratic Republic of the Congo; 10 Department of Virology, National Institute for Biomedical Research (INRB), Democratic Republic of the Congo, Kinshasa, Congo; 11 Service of Microbiology, Department of Medical Biology, Kinshasa teaching School of Medecine, Faculty of Medecine, University of Kinshasa, Democratic Republic of the Congo, Kinshasa, Congo; 12 IRD UMI 233, INSERM U1175, Université de Montpellier, Unité TransVIHMI, Montpellier, France; 13 EpiGreen, Paris, France; 14 Service d’Hygiène Hospitalière, Infectiovigilance et Prévention, Hospices Civils de Lyon, Épidémiologie, Lyon, France

**Keywords:** SARS-CoV-2, COVID-19 testing, Reverse transcriptase polymerase chain reaction, Serology, Africa.

## Abstract

**Objectives:**

In most African countries, confirmed COVID-19 case counts underestimate the number of new SARS-CoV-2 infection cases. We propose a multiplying factor to approximate the number of biologically probable new infections from the number of confirmed cases.

**Methods:**

Each of the first thousand suspect (or alert) cases recorded in South Kivu (DRC) between 29 March and 29 November 2020 underwent a RT-PCR test and an IgM and IgG serology. A latent class model and a Bayesian inference method were used to estimate (i) the incidence proportion of SARS-CoV-2 infection using RT-PCR and IgM test results, (ii) the prevalence using RT-PCR, IgM and IgG test results; and, (iii) the multiplying factor (ratio of the incidence proportion on the proportion of confirmed –RT-PCR+– cases).

**Results:**

Among 933 alert cases with complete data, 218 (23%) were RT-PCR+; 434 (47%) IgM+; 464 (~ 50%) RT-PCR+, IgM+, or both; and 647 (69%) either IgG + or IgM+. The incidence proportion of SARS-CoV-2 infection was estimated at 58% (95% credibility interval: 51.8–64), its prevalence at 72.83% (65.68–77.89), and the multiplying factor at 2.42 (1.95–3.01).

**Conclusions:**

In monitoring the pandemic dynamics, the number of biologically probable cases is also useful. The multiplying factor helps approximating it.

**Supplementary Information:**

The online version contains supplementary material available at 10.1186/s12874-023-02077-2.

## Introduction

According to the World Health Organisation (WHO), confirmed cases of Severe Acute Respiratory Syndrome Coronavirus 2 (SARS-CoV-2) infection are those with positive Nucleic Acid Amplification Test (NAAT) or those having both a positive SARS-CoV-2 Antigen-RDT (Rapid Diagnostic Test) and meeting some clinical criteria [1].

It is now recognized that, in Africa, confirmed case counts do not accurately reflect COVID-19 epidemic dynamics [2]. That underestimation of the number of new infections is illustrated by the contrast between a low number of reported cases and a high prevalence of anti-SARS-CoV-2 antibodies (an indicator of high virus circulation) [3]. In 2020, in South Kivu, a low number of reported confirmed cases (418 for 1526 alert –or suspect– cases) contrasted with a high seroprevalence (41.2%) among 359 healthcare workers [4]. According to some authors, that underestimation had two distinct causes: (1) a small portion of the population was tested; (2) a number of tests may have been negative in infected individuals [5, 6]. For economic or logistic reasons, the first cause is difficult to circumvent, whereas the second seems easier to deal with. In fact, estimates of tests’ sensitivity (Se) and specificity (Sp) are available as well as statistical methods that allow estimating the number of cases even with imperfect diagnostic tests [7–9].

In South Kivu, NAAT and antibody tests are currently available. Reverse Transcriptase-Polymerase Chain Reaction (RT-PCR) SARS-CoV-2 antigen detection test does not yield false-positive results, but false-negative results are possible because of poor sample quality or sample collection in very early or late stages of infection [10]. According to some authors, the Se of RT-PCR SARS-CoV-2 test (its ability to identify truly diseased individuals) was 55% [7], 68% [8], or 85% [9]; a large variability due to sample quality or sampling delay relative to infection. The antibody tests (i.e., rapid lateral flow tests for immunoglobulin M or G –IgM or IgG) that were available during the first wave of the pandemic could yield false-positive and false-negative results in detecting incident cases [11, 12]. Indeed, according to Kostoulas et al. [8], true positive results (Se) for either IgM or IgG during the first, second, and third week after COVID-19 symptom onset were obtained in 32, 75, and 92% of cases, respectively. However, the latter tests Sps were obviously lower than that of RT-PCR and ranged from 81 to 100% [7, 13].

The WHO definition of ‘confirmed cases’ based on RT-PCR results avoids count overestimation because of the very high Sp of RT-PCR (nearly 1). However, that definition gives only a lower limit for the real number of cases because of RT-PCR false-negative results. Serum IgM or IgG detection is not routinely used for case definition because of these tests’ false-negative and false-positive results. Besides, while RT-PCR and IgM tests are suitable to diagnose recent infections (incident cases), IgG test is rather suitable to assess past infections (prevalent cases). Given those elements, it seems useful to propose a way to obtain a better estimate of the number of new cases from the number of RT-PCR-confirmed cases.

This article proposes a method to obtain a factor by which the number of confirmed cases (precisely, RT-PCR + cases) may be multiplied to provide a better estimate of the number of new infections (i.e., the number of biologically probable cases).

In the following, we will: (i) estimate the incidence proportion and the prevalence of SARS-CoV-2 infection (here, the proportion of current or previous cases) in the first thousand COVID-19 alert cases in South Kivu using latent class model and a Bayesian inference method; and, (ii) propose an estimate of the multiplying factor that allows obtaining the number of biologically probable new SARS-CoV-2 infections from the number of confirmed cases.

## Materials and methods

### The study population

The data were extracted from the SARS-CoV-2 infection surveillance system in South Kivu (DRC) during the 2020 pandemic. The population of South Kivu is approximately 4,800,000 people who live in a 64,492 km² area. The study considered the first thousand alert cases recorded between March 29 and November 29, 2020. An alert case was defined as a person with signs suggestive of COVID-19 (fever, headache, breathing difficulties, asthenia… with or without loss of taste and smell) or a person who has been in contact with a person who tested positive for SARS-Cov-2 infection.

From each alert case, two samples were collected: a nasopharyngeal sample for RT-PCR test and a blood sample for IgM and IgG serology. In general, the recording of an alert case and sample collection took place the same day.

The serological test used ‘SARS-CoV-2 IgG/IgM Rapid Test Kit’ (Abbexa Ltd, Cambridge, UK). This test detects separately but on the same ‘cassette’ IgM and IgG antibodies against the virus. The tests (RT-PCR and serological test) were carried out in two centers: Kinshasa (March 29 to June 16, 2020) and Bukavu (June 17 to November 29, 2020).

A confirmed case was defined as an alert case with a positive RT-PCR test.

### Statistical analyses

#### Data presentation

Data presentation used 2 by 2 contingency tables for cross-tabulation of RT-PCR versus IgM test results (numbers and percentages), then for cross-tabulation of RT-PCR versus IgM and IgG test results (positive when IgM + or IgG+, negative when IgM– and IgG–). The information given by each cell of these tables depends on the proportion of infection cases, the Se, and the Sp of each test. For example, the number of alert cases positive on test A and test B is the sum of two numbers: (i) the number of true positive results on both tests (i.e., the number of alert cases multiplied by the proportion of infection cases and the Se of each test); and, (ii) the number of false positive results on both tests (i.e., the number of alert cases multiplied by the complement to 100% of the percentage of infected cases and the complement to 100% of the Sp of each test). This information is needed to estimate respectively the incidence proportion and the prevalence of SARS-CoV-2 infection using a latent class model and a Bayesian inference method.

#### The latent class model

In the latent class model, the infection status is considered unknown and the results of the diagnostic tests are used to estimate the proportion of infected cases and the performance (Se and Sp) of the tests. The model was built with the assumption that the RT-PCR and the antibody test results are independent conditionally on the infection status. In fact, this assumption is plausible because the two types of diagnostic tests (RT-PCR and IgM or IgG serology) have different biological mechanisms.

Two separate latent class models were used; one to estimate the incidence proportion (using RT-PCR and IgM serology) and the other to estimate the prevalence (using RT-PCR and IgM/IgG serology).

### The bayesian inference method

With two tests, the information provided by the observed data is not sufficient to estimate the proportion of infection cases and the performance of the tests in terms of Se and Sp. A Bayesian inference method was used to add prior knowledge on the Se of the RT-PCR and the Se and Sp of the serological tests to the observed data [14]. The Sp of RT-PCR was set to 100%. This implies the use of two latent classes (instead of four without this assumption).

Prior knowledge was extracted from the literature and summarized using prior distributions. Prior information on the performance of the tests was obtained from a search on PubMed with various combinations of keywords “COVID-19”, “diagnosis”, “performance”, “accuracy”, “test”, and “serological”. The retained articles were those that reported the performance of at least one of the tests (RT-PCR, IgG, and IgM). The excluded articles were those where the ‘gold standard’ was an imperfect diagnostic test, those that reported on pre-pandemic sera (to determine serologic test specificities), and those that used clinical or biological criteria to select the population. From the articles selected [7–9, 13], we extracted the smallest lower bound and the largest upper bound of the 95% confidence intervals (CoIs) of each test Se and Sp to derive prior intervals. When no confidence intervals were available, point estimates were used to derive the prior intervals (Table 1). Beta distributions were used as prior distributions with means equal to the centers of the corresponding prior intervals and standard deviations equal to the fourths of their ranges (Table 1). For the proportion of infection cases, a beta distribution with both parameters equal to one was used; this corresponds to a uniform distribution between 0 and 1.

**Table 1:** Prior knowledge on the sensitivities and specificities of the tests used for the diagnosis of SAR-Cov-2 infection

Test characteristic	Prior interval	Prior parameters of beta distributions	
		Alpha	Beta
RT-PCR sensitivity	50–91%	13.25	5.54
IgM test sensitivity ^a^	24–44%	30.18	58.58
IgM test specificity	94–100%	124.48	3.85
IgM and IgG test sensitivity ^b^	23–97%	3.61	2.40
IgM and IgG test specificity ^b^	81–100%	33.58	3.52

^a^ Sensitivity of the test in diagnosing recent infections

^b^ IgM and IgG test: positive result when IgM + or IgG+, negative result when IgM– and IgG–.

Gibbs sampling was used to obtain a sample of the posterior distribution of each parameter from which were derived a point estimate (median of the posterior distribution) and a 95% credibility interval (CrI, between quantiles 2.5% and 97.5% of the posterior distribution) [14].

Three sets of 60,000 values were sampled from the conditional posterior distribution of each of the three parameters of the model using three different sets of starting values for the parameters. These starting sets were chosen using the centres and the upper and lower bounds of the intervals of the literature data on test performance (Table 1). An interval was formed by the proportions of positive RT-PCR and IgG/IgM serology using the cross table of these two test results. The bounds and centre of this interval were used as a starting set for the proportion of infected people in the data. The convergence of the three Markov chains was evaluated by the Gelman index. The first 10,000 iterations of the three chains allowing to reach convergence were removed. The remaining 50,000 iterations of each of the three chains were put together to give point estimates (medians of the posterior distributions) and 95% credibility intervals (quantiles 2.5% and 97.5% of the posterior distributions) of the parameters.

### Estimating the multiplying factor

A sample of the posterior distribution of the multiplying factor was obtained by dividing each value of the posterior distribution sample of the incidence proportion of SARS-Cov-2 infection by the observed proportion of alert cases with positive RT-PCR test result. A point estimate of the factor and a 95% CrI were extracted from that sample (For more details, please see Additional files 1, 2 and 3).

In this work, qualitative variables were summarized by numbers and percentages in various modalities and quantitative variables by the mean, the standard deviation, the median, the first and third quartile.

All statistical analyses were performed with R software version 3.6.3 (2020-02-29, R Core Team (2020). R: A language and environment for statistical computing. R Foundation for Statistical Computing, Vienna, Austria. URL https://www.R-project.org/).

## Results

### Characteristics of the study population

The median age of the individuals in the alert population was 44 years and the standard deviation was nearly 17 years. This wide variability is also illustrated by a 25-year interquartile range (Q1 = 33 years and Q3 = 58 years).

Of the 929 individuals with no missing data for sex or diagnostic test results, (659) 71% were males. Among COVID-19 signs that motivated the alert, fever, dry cough, and fatigue were present in (595) 64%, 166 (18%), and 99 (11%) of alert cases, respectively.

### Observed diagnostic test results

Full RT-PCR and IgG/IgM test results were available for 933 out of the 1000 alert cases. According to these results, 218 (23%) cases were RT-PCR+; 434 (47%) IgM+; and 464 (50%) RT-PCR+, IgM+, or both. Besides, of these 933 cases, 647 (69%) were IgM + or IgG+ (Table 2).

**Table 2:** SARS-CoV-2 RT-PCR antigen test and serology results in the first thousand COVID-19 cases seen in South Kivu (DRC) in 2020

	RT-PCR negative	RT-PCR positive	Total	
IgM test result				
Negative	469 (50.27) ^a^	30 (3.22)	499 (53.49)	
Positive	246 (26.37)	188 (20.14)	434 (46.51)	
Total	715 (76.64)	218 (23.36)	933 (100)	
IgM and IgG test result ^b^				
Negative	267 (28.62) ^a^	19 (2.04)	286 (30.66)	
Positive	448 (48.02)	199 (21.32)	647 (69.34)	
Total	715 (76.64)	218 (23.36)	933 (100)	

^a^ Each value is expressed as number (percent of all available values)

^b^ IgM and IgG test: positive result when IgM + or IgG+, negative result when IgM– and IgG–.

### Estimates obtained with the latent class model

The incidence proportion of SARS-CoV-2 infection in the first thousand COVID-19 alert cases in South Kivu was estimated at 58% (95% CrI: 51.8–64) and the prevalence at 72.83% (95% CrI: 65.68–77.89). In other words, out of 100 alert subjects 58 were incident cases and about 73 were prevalent cases.

The RT-PCR SARS-CoV-2 antigen test sensitivity was estimated at 41.34% (95% CrI: 36.47–46.57). The IgM test sensitivity was estimated at 71.65% (95% CrI: 66.45–76.45) and its specificity at 96.93% (95% CrI: 92.75–99.11). These values were also observable in the posterior distributions of the parameters (see Additional file 4).

### Calculation of the multiplying factor

Of the 1000 alert subjects, 240 were predicted as confirmed cases versus 580 as probable cases. The factor for approximating the number of new biologically probable infections from the number of RT-PCR + subjects was 2.42 (95% CrI: 1.95–3.01) (Table 3). This means that the number of confirmed cases should be multiplied by 2.42 to yield an estimate of the number of new biologically probable infections.

**Table 3:** Calculation of the factor for approximating the number of new biologically probable infections from the number of RT-PCR + subjects in South Kivu (DRC).

Parameter	Median (95% credibility interval)
Expected number of RT-PCR + subjects among 1000 alert cases	240 (203–278)
Number of biologically probable new SARS-CoV-2 infections	580 (518–640)
Multiplying factor ^a^	2.42 (1.95–3.01)

^a^ Factor that allows obtaining the number of biologically probable new SARS-CoV-2 infections from the number of confirmed cases

## Discussion

This analysis of the first 1000 alert cases in South Kivu showed the strong underestimation of the number of new infections obtained by using the number of confirmed cases. Indeed, whereas RT-PCR test indicated 23% of confirmed cases, the incidence proportion as estimated by the latent class model indicated 58% of new infection cases and the estimated prevalence was 73%; i.e., the number of new infections would have been more than double of the number of confirmed cases. The high specificity of RT-PCR tests precludes overestimation and justifies its use to define confirmed cases. However, we might propose to join to the number of confirmed cases the number of biologically probable cases as obtained using the multiplying factor.

The impact of misclassification is well known in other contexts [15–18] and in infectious diseases [19] and has been taken into account in numerous studies. Either they use a method comparable to the one used here, based on estimating a multiplying factor to obtain the corrected value from the observed value [19], or they obtain the corrected value by a latent class model without giving a multiplying factor that can be used in another context. These methods are currently also used to estimate vaccine effectiveness [20–22]. Several previous studies have estimated the number of biologically probable cases using a latent class model to account for test imperfections [7–9, 23]. They reported comparable results in different geographical contexts and suggested for RT-PCR test higher sensitivity estimates than ours (55 to 85% vs. 36 to 47%). This discrepancy may arise from tests performed sometimes at late stages of the disease in this study context, which is supported by the high proportion of IgG + results.

Here, adding the information on IgG results would increase the estimate of the proportion of infection cases in the alert cases up to 73%. As the study concerned the 1000 alert cases identified during the first months of the pandemic, it is very probable that most infection cases have occurred during the study period and that the true incidence proportion among these first thousand alert cases lays between 58% (an underestimation due to late diagnoses) and 73% (an overestimation due to the potential inclusion of a small number of infection cases that had occurred before the study period). Logically, after the first year of the pandemic, the incidence proportion should be estimated using only the results of RT-PCR and IgM tests.

The early availability of new biological tests at the beginning of the pandemic justifies the caution in serological result interpretation. In particular, some authors have suspected a cross-reactivity between SARS-Cov-2 and other germ antigens already present in the African context [13, 24, 25]. The progressive improvement of serological tests make them useful complements to RT-PCR test whose sensitivity decreases at late stages of infection. Indeed the latent class model with Bayesian inference changed slightly the estimates of the proportion of infection cases by allowing for the imperfections of the tests. In fact, (i) the proportion of either RT-PCR + or IgM + subjects was 50%, whereas the estimate of the incidence proportion given by the model was 58%; and, (ii) the proportion of RT-PCR+, or IgM+, or IgG + subjects was 71%, whereas the estimate of the prevalence given by the model was 73%. This 58% incidence proportion is also found using the expression proposed by Sempos and Tian [26], but the contribution of the present study was to take into account the uncertainty on the performance of RT-PCR and quantify the multiplier factor in an African context.

The correction factor estimate allowing to approximate the number of probable new SARS-CoV-2 infections among the tested subjects, was based on the number of confirmed cases and was depended of the performance of the used tests. It can be transposed to other countries that used the same tests, regardless of the strength of the epidemic. The correction factor would have to be recalculated in countries that use other tests.

The method and the multiplication factor can be generalized to other African countries with different screening capacities, resources and epidemiological contexts. However, the multiplication factor will have to be recalculated if the tests used are different.

One limitation of this study may be the extrapolation of the incidence proportion found in the 933 subjects (who had diagnostic test results) to the rest of the first 1000 alerts (i.e. 67 subjects). But there was nothing to contradict the fact that these missing data were not random. Even if this bias exists, it will have little impact because these subjects represented only about 7% of the first 1000 alerts.

Another limitation was the unavailability of the delay between symptom onset and lab tests. Indeed, this delay may affect test results and subsequently the value of the multiplier. Furthermore, very few subjects were asymptomatic in our study, which limits subgroup analysis in this particular population.

## Conclusion

The present study confirmed that the incidence proportion of SARS-CoV-2 infection is underestimated when only RT-PCR positive subjects are counted. Thus, when dealing with changes in the dynamics of the pandemic, it would be useful to report the number of biologically probable cases along with the number of confirmed cases. The estimated multiplier may be used to approximate this number from the number of RT-PCR + cases in the African context. To further clarify the applicability of the proposed multiplier, this study should be supported by another one that aims to estimate the multiplier factor as a function of the time elapsed between the onset of COVID-19 symptoms and the carrying out of the diagnostic tests, and also in asymptomatic subjects.

### Electronic supplementary material

Below is the link to the electronic supplementary material.

Supplementary Material 1

Supplementary Material 2

Supplementary Material 3

Supplementary Material 4

## Data Availability

The datasets generated and/or analyzed during the current study are available from the corresponding author on reasonable request.

## List of abbreviations

CoIs: Confidence Intervals
CrI: Credibility Interval
DRC: Democratic Republic of the Congo
NAAT: Nucleic Acid Amplification Test
RDT: Rapid Diagnostic Test
Se: Sensitivity
Sp: Specificity
WHO: World Health Organization
