# Supplementary material for: Estimating the number of probable new SARS-CoV-2 infections among tested subjects from the number of confirmed cases
Source: BMC Med Res Methodol. 2023 Nov 17;23:272. doi: 10.1186/s12874-023-02077-2 (PMC10655282; doi:10.1186/s12874-023-02077-2)
Supplement: Supplementary file 2 — Supplementary Material 2 [file 12874_2023_2077_MOESM2_ESM.pdf]

```
# Latent class model script using RT-PCR and IgM


# Memory allocation for R software

memory.size()
memory.limit(size=5000000)
memory.size(max = NA)

# Workspace

setwd("D:\\CAMPUS\\LYON\\PROGRAMME\\M2\\stage\\5834 COVID_19_SUD_KIVU")

#packages

library(coda)

# Loading of data

don_source<-read.csv("source/donnnes au 06022021.csv",sep=";")

# Data with PCR, IgG and IgM results

don<-don_source[(don_source$resultatpcr=="Positif"|don_source$resultatpcr=="Néga
tif")&(don_source$resultatIgG=="Positif"|don_source$resultatIgG=="Négatif")&(don
_source$resultatIgM=="Positif"|don_source$resultatIgM=="Négatif"),]

# Reading the macro to estimate test performance and incidence proportion

source("programmes\\tt2_adapt.txt")

# Determination of the parameters of the a priori Beta distributions

mu.to.beta <-function(mu, sd)
{
        var <- sd^2
        alpha <-  - (mu * (var + mu^2 - mu))/var
        beta <- ((mu - 1) * (var + mu^2 - mu))/var
        list(alpha=alpha, beta=beta)
}

# Parameters of the a priori distribution of PCR sensitivity

dis_se1<-mu.to.beta(0.50+(0.91-0.50)/2,(0.91-0.50)/4)
dis_se1

# Parameters of the a priori distribution of IgM sensitivity

dis_se2<-mu.to.beta(0.24+(0.44-0.24)/2,(0.44-0.24)/4)
dis_se2

# Parameters of the a priori distribution of IgM specificity

dis_sp2<-mu.to.beta(0.94+(1-0.94)/2,(1-0.94)/4)
```

```
dis_sp2

# Table PCR IgM

don$pcr=ifelse(don$resultatpcr=="Positif","Positif",ifelse(don$resultatpcr=="Nég
atif","Négatif",NA))
don$igm=ifelse(don$resultatIgM=="Positif","Positif",ifelse(don$resultatIgM=="Nég
atif","Négatif",NA))

pcr_igm_table<-table(don$igm,don$pcr)
pcr_igm_table

# Estimation of test performance and incidence proportion

# Starting values lower bound
gibbs.sampler.out_inf<-tt2.gibbs(188,30,246,469,200,300,0.50,0.24,0.94,0.23,1,1,
13.25,5.54,30.1784,58.5816,124.4833,3.85,60000)

# Starting values upper bound
gibbs.sampler.out_sup<-tt2.gibbs(188,30,246,469,10,10,0.91,0.44,1,0.47,1,1,13.25
,5.54,30.1784,58.5816,124.4833,3.85,60000)

# Starting values centre
gibbs.sampler.out_cent<-tt2.gibbs(188,30,246,469,100,150,0.705,0.34,0.97,0.35,1,
1,13.25,5.54,30.1784,58.5816,124.4833,3.85,60000)


# Incidence proportion

prev.samp_inf=mcmc(gibbs.sampler.out_inf$prev.samp)
prev.samp_sup=mcmc(gibbs.sampler.out_sup$prev.samp)
prev.samp_cent=mcmc(gibbs.sampler.out_cent$prev.samp)
prev.samp0=mcmc.list(prev.samp_inf,prev.samp_sup,prev.samp_cent)

gelman.plot(prev.samp0)

# Selection of the first 10000 iterations

prev.samp_inf=mcmc(gibbs.sampler.out_inf$prev.samp[1:10000])
prev.samp_sup=mcmc(gibbs.sampler.out_sup$prev.samp[1:10000])
prev.samp_cent=mcmc(gibbs.sampler.out_cent$prev.samp[1:10000])
prev.samp1=mcmc.list(prev.samp_inf,prev.samp_sup,prev.samp_cent)

plot(prev.samp1)

gelman.plot(prev.samp1)
gelman.diag(prev.samp1)

# Remaining iterations

prev.samp_inf=mcmc(gibbs.sampler.out_inf$prev.samp[10001:60000])
prev.samp_sup=mcmc(gibbs.sampler.out_sup$prev.samp[10001:60000])
prev.samp_cent=mcmc(gibbs.sampler.out_cent$prev.samp[10001:60000])
prev.samp2=mcmc.list(prev.samp_inf,prev.samp_sup,prev.samp_cent)
```

```
plot(prev.samp2,density= FALSE,ylab="Pi")
plot(prev.samp2,trace= FALSE,xlab="",ylab="Density (%)")

gelman.plot(prev.samp2)
gelman.diag(prev.samp2)

autocorr.plot(prev.samp2)
autocorr.diag(prev.samp2)

raftery.diag(prev.samp2)

summary(prev.samp2)

# PCR sensitivity

sens1.samp_inf=mcmc(gibbs.sampler.out_inf$sens1.samp)
sens1.samp_sup=mcmc(gibbs.sampler.out_sup$sens1.samp)
sens1.samp_cent=mcmc(gibbs.sampler.out_cent$sens1.samp)
sens1.samp0=mcmc.list(sens1.samp_inf,sens1.samp_sup,sens1.samp_cent)

gelman.plot(sens1.samp0)

# Selection of the first 10000 iterations

sens1.samp_inf=mcmc(gibbs.sampler.out_inf$sens1.samp[1:10000])
sens1.samp_sup=mcmc(gibbs.sampler.out_sup$sens1.samp[1:10000])
sens1.samp_cent=mcmc(gibbs.sampler.out_cent$sens1.samp[1:10000])
sens1.samp1=mcmc.list(sens1.samp_inf,sens1.samp_sup,sens1.samp_cent)

plot(sens1.samp1)
gelman.plot(sens1.samp1)
gelman.diag(sens1.samp1)

# Remaining iterations

sens1.samp_inf=mcmc(gibbs.sampler.out_inf$sens1.samp[10001:60000])
sens1.samp_sup=mcmc(gibbs.sampler.out_sup$sens1.samp[10001:60000])
sens1.samp_cent=mcmc(gibbs.sampler.out_cent$sens1.samp[10001:60000])
sens1.samp2=mcmc.list(sens1.samp_inf,sens1.samp_sup,sens1.samp_cent)

plot(sens1.samp2,density= FALSE,ylab="se-RT-PCR")
plot(sens1.samp2,trace= FALSE,xlab="",ylab="Density (%)")

gelman.plot(sens1.samp2)
gelman.diag(sens1.samp2)

autocorr.plot(sens1.samp2)
autocorr.diag(sens1.samp2)

raftery.diag(sens1.samp2)

summary(sens1.samp2)
```

```
# IgM sensitivity

sens2.samp_inf=mcmc(gibbs.sampler.out_inf$sens2.samp)
sens2.samp_sup=mcmc(gibbs.sampler.out_sup$sens2.samp)
sens2.samp_cent=mcmc(gibbs.sampler.out_cent$sens2.samp)
sens2.samp0=mcmc.list(sens2.samp_inf,sens2.samp_sup,sens2.samp_cent)

gelman.plot(sens2.samp0)

# Selection of the first 10000 iterations

sens2.samp_inf=mcmc(gibbs.sampler.out_inf$sens2.samp[1:10000])
sens2.samp_sup=mcmc(gibbs.sampler.out_sup$sens2.samp[1:10000])
sens2.samp_cent=mcmc(gibbs.sampler.out_cent$sens2.samp[1:10000])
sens2.samp1=mcmc.list(sens2.samp_inf,sens2.samp_sup,sens2.samp_cent)

plot(sens2.samp1)

gelman.plot(sens2.samp1)
gelman.diag(sens2.samp1)

# Remaining iterations

sens2.samp_inf=mcmc(gibbs.sampler.out_inf$sens2.samp[10001:60000])
sens2.samp_sup=mcmc(gibbs.sampler.out_sup$sens2.samp[10001:60000])
sens2.samp_cent=mcmc(gibbs.sampler.out_cent$sens2.samp[10001:60000])
sens2.samp2=mcmc.list(sens2.samp_inf,sens2.samp_sup,sens2.samp_cent)

plot(sens2.samp2,density= FALSE,ylab="se-IgM")
plot(sens2.samp2,trace= FALSE,xlab="",ylab="Density (%)")

gelman.plot(sens2.samp2)
gelman.diag(sens2.samp2)

autocorr.plot(sens2.samp2)
autocorr.diag(sens2.samp2)

raftery.diag(sens2.samp2)

summary(sens2.samp2)

# IgM specificity

spec2.samp_inf=mcmc(gibbs.sampler.out_inf$spec2.samp)
spec2.samp_sup=mcmc(gibbs.sampler.out_sup$spec2.samp)
spec2.samp_cent=mcmc(gibbs.sampler.out_cent$spec2.samp)
spec2.samp0=mcmc.list(spec2.samp_inf,spec2.samp_sup,spec2.samp_cent)

gelman.plot(spec2.samp0)

# Selection of the first 10000 iterations

spec2.samp_inf=mcmc(gibbs.sampler.out_inf$spec2.samp[1:10000])
spec2.samp_sup=mcmc(gibbs.sampler.out_sup$spec2.samp[1:10000])
```

```
spec2.samp_cent=mcmc(gibbs.sampler.out_cent$spec2.samp[1:10000])
spec2.samp1=mcmc.list(spec2.samp_inf,spec2.samp_sup,spec2.samp_cent)

plot(spec2.samp1)

gelman.plot(spec2.samp1)
gelman.diag(spec2.samp1)

# Remaining iterations

spec2.samp_inf=mcmc(gibbs.sampler.out_inf$spec2.samp[10001:60000])
spec2.samp_sup=mcmc(gibbs.sampler.out_sup$spec2.samp[10001:60000])
spec2.samp_cent=mcmc(gibbs.sampler.out_cent$spec2.samp[10001:60000])
spec2.samp2=mcmc.list(spec2.samp_inf,spec2.samp_sup,spec2.samp_cent)

plot(sens2.samp2,density= FALSE,ylab="sp-IgM")
plot(sens2.samp2,trace= FALSE,xlab="",ylab="Density (%)")

gelman.plot(spec2.samp2)
gelman.diag(spec2.samp2)

autocorr.plot(spec2.samp2)
autocorr.diag(spec2.samp2)

raftery.diag(spec2.samp2)

summary(spec2.samp2)

# Number of infected IgM positive, PCR negative

y3.samp_inf=mcmc(gibbs.sampler.out_inf$y3.samp)
y3.samp_sup=mcmc(gibbs.sampler.out_sup$y3.samp)
y3.samp_cent=mcmc(gibbs.sampler.out_cent$y3.samp)
y3.samp0=mcmc.list(y3.samp_inf,y3.samp_sup,y3.samp_cent)

gelman.plot(y3.samp0)

# Selection of the first 10000 iterations

y3.samp_inf=mcmc(gibbs.sampler.out_inf$y3.samp[1:10000])
y3.samp_sup=mcmc(gibbs.sampler.out_sup$y3.samp[1:10000])
y3.samp_cent=mcmc(gibbs.sampler.out_cent$y3.samp[1:10000])
y3.samp1=mcmc.list(y3.samp_inf,y3.samp_sup,y3.samp_cent)

plot(y3.samp1)

gelman.plot(y3.samp1)
gelman.diag(y3.samp1)

# Remaining iterations

y3.samp_inf=mcmc(gibbs.sampler.out_inf$y3.samp[10001:60000])
y3.samp_sup=mcmc(gibbs.sampler.out_sup$y3.samp[10001:60000])
y3.samp_cent=mcmc(gibbs.sampler.out_cent$y3.samp[10001:60000])
```

```
y3.samp2=mcmc.list(y3.samp_inf,y3.samp_sup,y3.samp_cent)

plot(y3.samp2)


gelman.plot(y3.samp2)
gelman.diag(y3.samp2)

autocorr.plot(y3.samp2)
autocorr.diag(y3.samp2)

raftery.diag(y3.samp2)

summary(y3.samp2)

# Number of infected IgM and PCR negative

y4.samp_inf=mcmc(gibbs.sampler.out_inf$y4.samp)
y4.samp_sup=mcmc(gibbs.sampler.out_sup$y4.samp)
y4.samp_cent=mcmc(gibbs.sampler.out_cent$y4.samp)
y4.samp0=mcmc.list(y4.samp_inf,y4.samp_sup,y4.samp_cent)

gelman.plot(y4.samp0)

# Selection of the first 10000 iterations

y4.samp_inf=mcmc(gibbs.sampler.out_inf$y4.samp[1:10000])
y4.samp_sup=mcmc(gibbs.sampler.out_sup$y4.samp[1:10000])
y4.samp_cent=mcmc(gibbs.sampler.out_cent$y4.samp[1:10000])
y4.samp1=mcmc.list(y4.samp_inf,y4.samp_sup,y4.samp_cent)

plot(y4.samp1)

gelman.plot(y4.samp1)
gelman.diag(y4.samp1)

# Remaining iterations

y4.samp_inf=mcmc(gibbs.sampler.out_inf$y4.samp[10001:60000])
y4.samp_sup=mcmc(gibbs.sampler.out_sup$y4.samp[10001:60000])
y4.samp_cent=mcmc(gibbs.sampler.out_cent$y4.samp[10001:60000])
y4.samp2=mcmc.list(y4.samp_inf,y4.samp_sup,y4.samp_cent)

plot(y4.samp2)

gelman.plot(y4.samp2)
gelman.diag(y4.samp2)

autocorr.plot(y4.samp2)
autocorr.diag(y4.samp2)

raftery.diag(y4.samp2)

summary(y4.samp2)
```

```
# incidence

inci_inf<-mcmc(rbinom(50000,1000,prev.samp_inf))
inci_sup<-mcmc(rbinom(50000,1000,prev.samp_sup))
inci_cent<-mcmc(rbinom(50000,1000,prev.samp_cent))
inci<-mcmc.list(inci_inf,inci_sup,inci_cent)

summary(inci)

# Number of RT-PCR positive subjects

pos_pcr_inf<-mcmc(rbinom(50000,580,sens1.samp_inf))
pos_pcr_sup<-mcmc(rbinom(50000,580,sens1.samp_sup))
pos_pcr_cent<-mcmc(rbinom(50000,580,sens1.samp_cent))
pos_pcr<-mcmc.list(pos_pcr_inf,pos_pcr_sup,pos_pcr_cent)

summary(pos_pcr)



# Factor to approximate the incidence from the number of RT-PCR positive
subjects

coef_inf<-inci_inf/pos_pcr_inf
coef_sup<-inci_sup/pos_pcr_sup
coef_cent<-inci_cent/pos_pcr_cent
coef<-mcmc.list(coef_inf,coef_sup,coef_cent)

summary(coef)


plot(coef,density= FALSE,ylab="MF");
plot(coef,trace= FALSE,xlab="",ylab="Density (%)")
gelman.plot(coef)
gelman.diag(coef)

# Summary of Markov chains


plot(sens1.samp2,density= FALSE,ylab="se-RT-PCR",cex.axis=1.5,cex.lab=1.5)
plot(sens1.samp2,trace= FALSE,xlab="",ylab="Density
(%)",cex.axis=1.5,cex.lab=1.5)
gelman.plot(sens1.samp0,cex.axis=1.5,cex.lab=1.5)

plot(sens2.samp2,density= FALSE,ylab="se-IgM",cex.axis=1.5,cex.lab=1.5)
plot(sens2.samp2,trace= FALSE,xlab="",ylab="Density
(%)",cex.axis=1.5,cex.lab=1.5)
gelman.plot(sens2.samp0,cex.axis=1.5,cex.lab=1.5)

plot(spec2.samp2,density= FALSE,ylab="sp-IgM",cex.axis=1.5,cex.lab=1.5)
plot(spec2.samp2,trace= FALSE,xlab="",ylab="Density
(%)",cex.axis=1.5,cex.lab=1.5)
gelman.plot(spec2.samp0,cex.axis=1.5,cex.lab=1.5)
```

```
plot(prev.samp2,density= FALSE,ylab="Pi",cex.axis=1.5,cex.lab=1.5)
plot(prev.samp2,trace= FALSE,xlab="",ylab="Density
(%)",cex.axis=1.5,cex.lab=1.5)
gelman.plot(prev.samp0,cex.axis=1.5,cex.lab=1.5)
```
