# Supplementary material for: Estimating the number of probable new SARS-CoV-2 infections among tested subjects from the number of confirmed cases
Source: BMC Med Res Methodol. 2023 Nov 17;23:272. doi: 10.1186/s12874-023-02077-2 (PMC10655282; doi:10.1186/s12874-023-02077-2)
Supplement: Supplementary file 3 — Supplementary Material 3 [file 12874_2023_2077_MOESM3_ESM.pdf]

A. Likelihood of the model

The likelihood of the model combining RT-PCR and IgM serology (under the hypothesis of conditional independence of the two tests), denoted L , is defined by :

$$L = P(n_1, n_2, n_3, n_4 | se_1, se_2, sp_1, sp_2, \pi) \propto$$

$$(se_1 \times se_2 \times \pi + (1 - sp_1) \times (1 - sp_2) \times (1 - \pi))^{n_1}$$

$$\times ((1 - se_1) \times (1 - se_2) \times \pi + sp_1 \times sp_2 \times (1 - \pi))^{n_2}$$

$$\times (se_1 \times (1 - se_2) \times \pi + (1 - sp_1) \times sp_2 \times (1 - \pi))^{n_3}$$

$$\times ((1 - se_1) \times se_2 \times \pi + sp_1 \times (1 - sp_2) \times (1 - \pi))^{n_4}$$

Where π is the proportion of infected subjects, se_1 the sensitivity of RT-PCR, se_2 the sensitivity of IgM serology, sp_1 the specificity of RT-PCR, sp_2 the specificity of IgM serology and n_1, n_2, n_3, n_4 are respectively the number of subjects with a positive result for the both tests, with a negative result for the both tests, with a positive RT-PCR and a negative IgM serology , with a negative RT-PCR and a positive IgM serology. Thus the augmented likelihood of the corresponding latent class model, denoted LE , is defined by :

$$LE = P(n_1, n_2, n_3, n_4, y_1, y_2, y_3, y_4 | se_1, se_2, sp_2, \pi) \propto$$

$$(\pi \times se_1 \times se_2)^{y_1}$$

$$\times (\pi \times (1 - se_1) \times (1 - se_2))^{y_2}$$

$$\times (\pi \times se_1 \times (1 - se_2))^{y_3}$$

$$\times (\pi \times (1 - se_1) \times se_2)^{y_4}$$

$$\times ((1 - \pi) \times (1 - sp_1) \times (1 - sp_2))^{n_1 - y_1}$$

$$\times ((1 - \pi) \times sp_1 \times sp_2)^{n_2 - y_2}$$

$$\times ((1 - \pi) \times (1 - sp_1) \times sp_2)^{n_3 - y_3}$$

$$\times ((1 - \pi) \times sp_1 \times (1 - sp_2))^{n_4 - y_4}$$

Where y_1, y_2, y_3, y_4 are the latent numbers corresponding to the number of infected subjects among respectively the n_1, n_2, n_3, n_4 subjects. The specificity of RT-PCR was set at 100%, which implies that $n_1 - y_1 = 0$ and $n_3 - y_3 = 0$. After simplification, we obtain:

$$LE = P(n_1, n_2, n_3, n_4, y_2, y_4 | se_1, se_2, sp_2, \pi) \propto$$

$$(\pi)^{n_1 + y_2 + n_3 + y_4} \times (1 - \pi)^{n_2 - y_2 + n_4 - y_4}$$

$$\times (se_1)^{n_1 + n_3} \times (1 - se_1)^{y_2 + y_4}$$

$$\times (se_2)^{n_1 + y_4} \times (1 - se_2)^{y_2 + n_3}$$

$$\times (sp_2)^{n_2 - y_2} \times (1 - sp_2)^{n_4 - y_4}$$

A. Multiplying factor (MF)

$$MF = \frac{proportion\ infected}{proportion\ of\ positive\ RT\text{−}PCR}$$

$$MF = \frac{\pi}{\frac{z}{N}} = \frac{\pi}{\frac{(Se_{RT-PCR})\times A}{N}} = \frac{\pi}{\frac{(Se_{RT-PCR})\times \pi \times N}{N}}$$

$$MF = \frac{1}{Se_{RT-PCR}}$$

Where π is the proportion infected, z the number of RT-PCR positive subjects, N total count, A the number of subjects infected and Se_{RT-PCR} the sensitivity of RT-PCR.
