# Supplementary material for: Estimating the number of probable new SARS-CoV-2 infections among tested subjects from the number of confirmed cases
Source: BMC Med Res Methodol. 2023 Nov 17;23:272. doi: 10.1186/s12874-023-02077-2 (PMC10655282; doi:10.1186/s12874-023-02077-2)
Supplement: Supplementary file 4 — Supplementary Material 4 [file 12874_2023_2077_MOESM4_ESM.pdf]

Figure S1 : Summary of Markov chains of the latent class model using RT-PCR and IgM

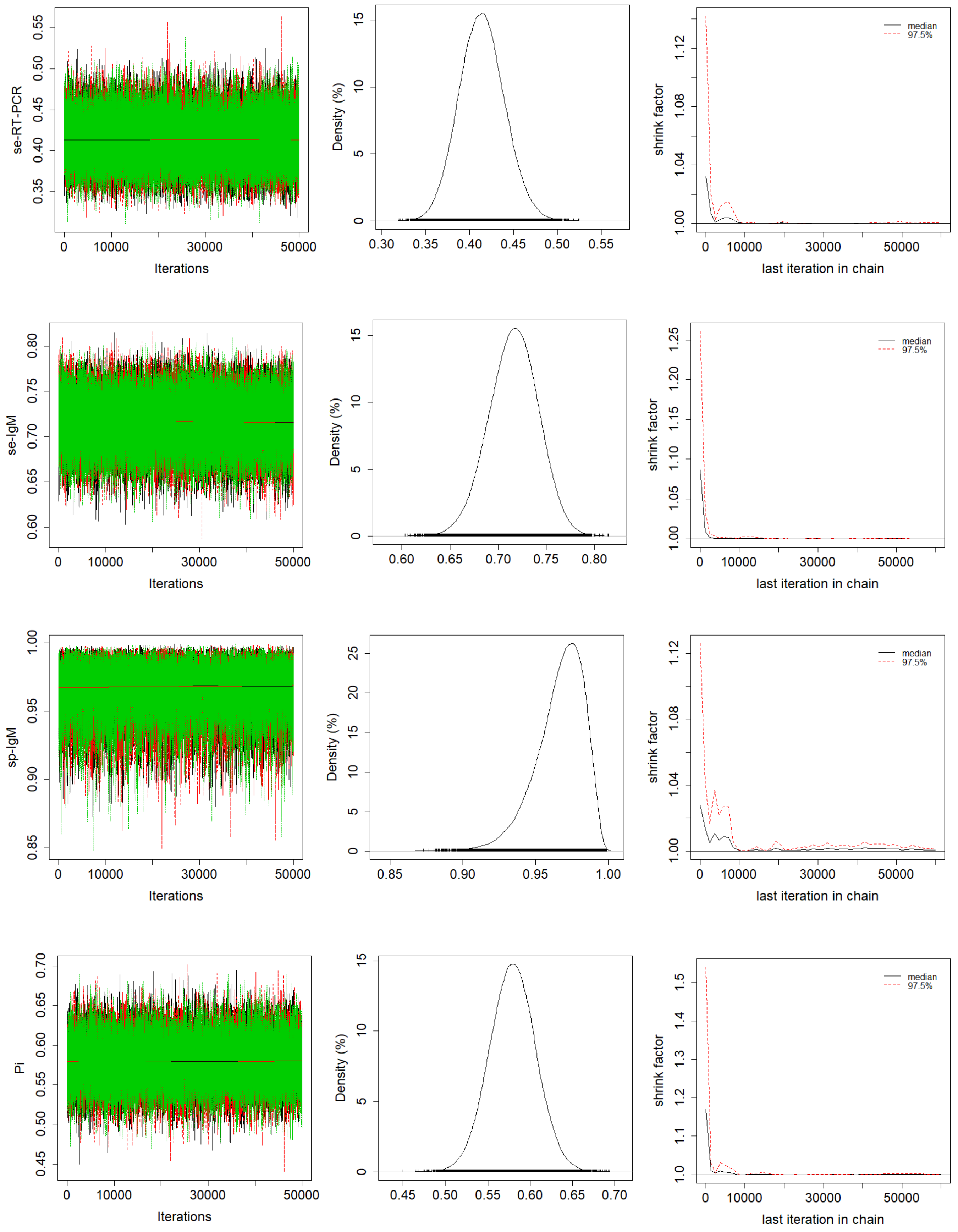

se-RT-PCR : RT-PCR (Reverse Transcriptase-Polymerase Chain Reaction) sensitivity
se-IgM : IgM (Class M immunoglobulin) sensitivity
sp-IgM : IgM (Class M immunoglobulin) specificity
Pi : Incidence proportion
